# PD-L1/PD-1 Pattern of Expression Within the Bone Marrow Immune Microenvironment in Smoldering Myeloma and Active Multiple Myeloma Patients

**DOI:** 10.3389/fimmu.2020.613007

**Published:** 2021-01-08

**Authors:** Federica Costa, Rosanna Vescovini, Valentina Marchica, Paola Storti, Laura Notarfranchi, Benedetta Dalla Palma, Denise Toscani, Jessica Burroughs-Garcia, Maria Teresa Catarozzo, Gabriella Sammarelli, Nicola Giuliani

**Affiliations:** ^1^ Department of Medicine and Surgery, University of Parma, Parma, Italy; ^2^ Hematology, “Azienda Ospedaliero-Universitaria di Parma”, Parma, Italy

**Keywords:** multiple myeloma, programmed cell death protein-1, programmed cell death protein-ligand 1, smoldering myeloma, immune checkpoint

## Abstract

**Background:**

The PD-1/PD-L1 axis has recently emerged as an immune checkpoint that controls antitumor immune responses also in hematological malignancies. However, the use of anti-PD-L1/PD-1 antibodies in multiple myeloma (MM) patients still remains debated, at least in part because of discordant literature data on PD-L1/PD-1 expression by MM cells and bone marrow (BM) microenvironment cells. The unmet need to identify patients which could benefit from this therapeutic approach prompts us to evaluate the BM expression profile of PD-L1/PD-1 axis across the different stages of the monoclonal gammopathies.

**Methods:**

The PD-L1/PD-1 axis was evaluated by flow cytometry in the BM samples of a total cohort of 141 patients with monoclonal gammopathies including 24 patients with Monoclonal Gammopathy of Undetermined Significance (MGUS), 38 patients with smoldering MM (SMM), and 79 patients with active MM, including either newly diagnosed or relapsed-refractory patients. Then, data were correlated with the main immunological and clinical features of the patients.

**Results:**

First, we did not find any significant difference between MM and SMM patients in terms of PD-L1/PD-1 expression, on both BM myeloid (CD14^+^) and lymphoid subsets. On the other hand, PD-L1 expression by CD138^+^ MM cells was higher in both SMM and MM as compared to MGUS patients. Second, the analysis on the total cohort of MM and SMM patients revealed that PD-L1 is expressed at higher level in CD14^+^CD16^+^ non-classical monocytes compared with classical CD14^+^CD16^−^ cells, independently from the stage of disease. Moreover, PD-L1 expression on CD14^+^ cells was inversely correlated with BM serum levels of the anti-tumoral cytokine, IL-27. Interestingly, relapsed MM patients showed an inverted CD4^+^/CD8^+^ ratio along with high levels of pro-tumoral IL-6 and a positive correlation between %CD14^+^PD-L1^+^ and %CD8^+^PD-1^+^ cells as compared to both SMM and newly diagnosed MM patients suggesting a highly compromised immune-compartment with low amount of CD4^+^ effector cells.

**Conclusions:**

Our data indicate that SMM and active MM patients share a similar PD-L1/PD-1 BM immune profile, suggesting that SMM patients could be an interesting target for PD-L1/PD-1 inhibition therapy, in light of their less compromised and more responsive immune-compartment.

## Introduction

Immune dysregulation is one of the hallmarks of Multiple myeloma (MM) (1). The interaction between MM cells and bone marrow (BM) microenvironment cells, together with hypoxic condition, create a tumor-permissive niche, characterized by an impaired dendritic cell (DC) differentiation and maturation, high levels of myeloid derived suppressor cells (MDSCs), and regulatory T cells, along with an unbalanced ratio of T helper (Th)1/Th2 cells and dysfunctional natural killer (NK) cells (1). Targeting the BM immune cells thus represents an effective strategy to prevent tumor progression.

The programmed cell death protein (PD)-1/PD-ligand (PD-L1) axis has recently emerged as a central immune checkpoint that controls antitumor immune responses against both solid tumours and hematologic malignancies.

PD-1 is expressed on the surface of exhausted T and B cells and is involved in the maintenance of peripheral self-tolerance, through the binding to its ligands PD-L1/PD-L2 mainly expressed on antigen presenting cells as macrophages and DCs (2). However, PD-L1/PD-1 surface expression is increased in several cancers, thus representing a mechanism of tumor escape from immune response (2).

The use of therapeutic agents blocking this pathway has showed impressive disease regression in several cancers, such as melanoma, non-small-cell lung cancer (NSCLC) and Hodgkin disease (3, 4).

Despite the encouraging results in MM pre-clinical models showing the efficacy of PD-1 blockade (5–8), early clinical studies on PD-1/PD-L1 inhibitors showed a minimal clinically meaningful anti-MM activity as single agents (9, 10), with only one complete response out of 27 MM patients and stable disease in 63% of the patient cohort treated with nivolumab (9). In addition, FDA put on hold all the clinical trials using anti–PD-1/PD-L1 antibodies in combination with other therapeutic agents, as the immunomodulatory drugs (IMiDs), in MM because of severe adverse events (https://www.fda.gov/Drugs/DrugSafety/ucm574305.htm).

It thus remains unclear which are the mechanisms behind anti–PD-1/PD-L1 lack of response, and which is the best patient subset that could benefit from this therapy. Indeed, discordant literature data have been reported on PD-1/PD-L1 expression in MM cells and BM niche cells (7, 8, 11–14). Some groups described a significant increase of PD-L1 expression on plasmacells (PC) from MM patients (including both newly diagnosed and relapsed myeloma) as compared with monoclonal gammopathies of undetermined significance (MGUS) and healthy donors (HDs) (7, 11, 12), while others did not find any significant difference (13, 15). Same discrepancies were reported on PD-1 expression in the T cell compartment of HDs and MM patients (7, 13). Limited literature data are instead available on PD-L1/PD-1 expression on myeloid compartments in relation with the disease stage, which anyway show no differences in terms of PD-L1 expression on MDSCs between newly diagnosed (MMD) and relapsed (MMR) MM patients, without comparing the results with healthy controls or patients at early stages of disease (7, 16). More recently, a study from Bailur et al. (14) analyzed BM microenvironment cells from HDs, MGUS and MM patients by mass-cytometry, which interestingly revealed an increased PD-L1 expression on bulk myeloid cells only between HDs and MM patients, with no differences between MGUS and MM (14).

Finally, few data have been reported on asymptomatic patients with smoldering MM (SMM) (15) thus suggesting the need to better define PD-1/PD-L1 distribution in the tumour microenvironment of patients with monoclonal gammopathies at different stages of disease.

The aim of this study was to analyse PD-1/PD-L1 expression in the BM niche of patients with MGUS, asymptomatic SMM or active disease, including MMD and MMR patients, and to further investigate a possible correlation with both clinical parameters, as cytogenetic abnormalities and bone disease, and serum levels of soluble factors involved in MM pathophysiology and immune response.

## Materials and Methods

### Patient Samples

BM aspirate were sequentially collected from a total cohort of 141 patients, including 24 MGUS (10 F, 14 M; median age 69, range 44–89), 38 SMM (16 F, 22 M; median age 68, range 36–87), 43 MMD (20 F, 23 M; median age 76, range 46–90, ISS: 13 = I, 10 = II, 19 = III), 36 MMR (16 F, 20 M; median age 75, range 59–89; ISS: 9 = I, 11 = II, 16 = III) (summarized in **Table 1**), admitted to the Hematology Unit of Parma Hospital.

**Table 1:** Summary of patient clinical characteristics.

Diagnosis	Nr	Sex		Median Age (range)	ISS			Light chain		Median PC % (range)	Osteolysis			High cytogenetic risk
		M	F		I	II	III	κ	λ		Yes	No	N/A	
**MGUS**	24	14	10	69 (44–89)				20	4	7 (4–9)	–	–		
**SMM**	38	22	16	68 (36–87)				26	13	13 (0.3–40)	–	–		
**MMD**	43	23	20	76 (46–90)	14	10	19	24	19	50 (20–95)	34	6	3	yes (56%)
**MMR**	36	20	16	75 (59–89)	9	11	16	20	16	40 (12–100)	26	7	3	yes (68%)

MGUS, monoclonal gammopathy of undetermined significance; SMM, Smoldering MM; MM, Multiple Myeloma; MMD, Newly Diagnosed MM; MMR, Relapsed MM; Nr, number; F, female; M, male; ISS, International Staging System; PC%, percentage of plasma cells evaluated by bone biopsy; N/A, not available; High risk, defined by presence of del(17p) and/or IGH translocations t(4;14)/t(14;16)/t(16;20) and/or gain(1q) and/or del(1p).

Clinical details about the treatments previously administered to MMR patients and data on the eventual refractoriness are specified in **Table 2**.

**Table 2:** Details on therapeutic lines and information on refractoriness in MMR patients.

Diagnosis	Previous Treatments			Therapeutic lines received (n.)	Refractoriness	
	Thal/IMiDs (Y/N)	PIs (Y/N)	ASCT (Y/N)		PIs (Y/N)	IMiDs (Y/N)
MMR1	Y	Y	N	2	N	Y
MMR2	–	–	–	1	NV	NV
MMR3	Y	Y	N	2	N	Y
MMR4	N	Y	N	1	Y	
MMR5	Y	Y	Y	1	N	N
MMR6	Y	Y	N	>3	N	N
MMR7	Y	N	N	2		Y
MMR8	N	Y	N	1	Y	
MMR9	N	Y	N	1	Y	
MMR10	N	Y	N	1	N	
MMR11	N	Y	N	1	Y	
MMR12	N	Y	N	1	Y	
MMR13	N	Y	N	1	N	
MMR14	Y	Y	Y	1	N	N
MMR15	Y	Y	Y	>3	Y	Y
MMR16	N	Y	N	1	N	
MMR17	N	Y	N	1	N	
MMR18	Y	N	N	1		Y
MMR19	Y	Y	N	2	N	N
MMR20	Y	Y	N	3	N	N
MMR21	N	Y	N	1	Y	
MMR22	Y	Y	Y	3	N	N
MMR23	N	Y	N	1	Y	
MMR24	Y	Y	N	3	N	Y
MMR25	N	Y	N	1	N	
MMR26	Y	N	N	1		N
MMR27	Y	Y	Y	1	N	N
MMR28	Y	Y	Y	1	N	N
MMR29	N	Y	N	1	N	
MMR30	N	Y	N	1	N	
MMR31	Y	Y	Y	>3	Y	Y
MMR32	Y	Y	Y	1	N	N
MMR33	Y	Y	Y	>3	Y	Y
MMR34	Y	N	N	2		Y
MMR35	Y	Y	N	3	Y	Y
MMR36	Y	Y	N	>3	Y	Y

MMR, relapsed/refractory multiple myeloma; Thal, Thalidomide; IMiDs, immunomodulatory drugs; PIs, proteasome inhibitors; ASCT, autologous stem cell transplantation; Y, Yes; N, Not; NV, not evaluable (patient enrolled in a blind study, not yet concluded); n., number.

Patient samples were obtained after informed consent, according to the Declaration of Helsinki. The study was approved by the Institutional Ethical Review Board of our Hospital.

The presence of osteolytic lesions in MM patients were detected by using X-ray skeletal survey as the first imaging procedure and alternatively a low-dose computed tomography scan or computed tomography/positron emission tomography, as indicated by the International Myeloma Working Group.

78% of MMD and 72% of MMR were osteolytic.

SMM patients were stratified according to the two main different score systems: Mayo-Clinic 2/20/20 score and PETHEMA score. Patients with active MM were classified according to the cytogenetic features, into high risk (HR) and standard risk (SR), based respectively on the presence or absence of IGH translocations t(4;14)/t(14;16)/t(16;20) and/or del(17p) and/or gain(1q) and/or del(1p) (17), assessed by FISH.

BM Mononuclear cells (MNCs) were isolated after Ficoll gradient stratification and analyzed by flow-cytometry.

BM plasma and serum were collected from heparin tubes by centrifugation and stored at −20°C until the analysis.

### Flow Cytometry Analysis

Immunephenotype analysis were performed on fresh collected BM MNCs, pre-incubated for 10 min with the staining buffer added with 10% of mouse serum, and then stained using different panels with the following monoclonal antibodies (mAbs) conjugated with chromophores (all purchased from BD Biosciences, Franklin Lakes, NL, USA): Anti-CD138 R-phycoerythrin (PE) (cod.552026), anti-CD14 fluorescein isothiocyanate (FITC) (cod.555397), anti-CD274 Allophycocyanin (APC) (cod.563741), anti-CD3 FITC (cod.555339), anti-CD4 APC (cod.555349), anti-CD8 APC (cod.555369), anti-CD279 PE (cod.557946), anti-CD16 PE (cod.555407), anti-CX3CR1 PE (cod. 565796). 7-Amino Actinomycin D (7-AAD) was used as cell viability die, in all the staining panels, according to manufacturer protocols. We used the Fluorescence minus one (FMO) controls to set PD-L1 and PD-1 specific gates.

The analysis was performed on a two-laser FACSCalibur instrument (BD Biosciences) using CellQuest software (BD Biosciences).

The applied gating strategy was based on a first live gating on 7-AAD negative cells, followed by a FSC/SSC scatter gate to identify the cells of interest according to their relative size and complexity, while removing debris and cell fragments.

PC were identified as CD138^+^, total monocytes as CD14^+^, then divided into CD16^−^ and CD16^+^; % of PD-L1^+^ cells and PD-L1 MFI were then evaluated on these cell populations. Total T cells (CD3^+^) were classified into CD4^+^ and CD8^+^ subsets and then analyzed for PD-1 expression (both % and MFI). Data on monocytes and T cells were normalized on the CD138^−^ fraction. A representative gating strategy is showed in **Supplementary Figure 1**.

### Cell Culture

For some experiments, BM MNCs were coltured into 12-well plates (5 × 10^5^ cells/ml) in RPMI 1640 medium (GIBCO^®^ by Life Technologies, MA, USA) added of 20% of fetal bovine serum (FBS), in the presence or absence of IL-27 (50 ng/ml, R&D System), IFN-γ (100 UI/ml, Sigma Aldrich), or IL-6 (20 ng/ml, Thermo Fisher Scientific) for 96 h. Cells were collected and analyzed by flow-cytometry after 24 h and at the end of colture period, in order to evaluate PD-L1 expression on both CD138^+^ cells and CD14^+^ monocytes after short and long term treatment.

### ELISA

BM serum levels of IFN-γ, IL-6, IL-10, and IL-27 were measured by high-sensitivity ELISA, using commercially available kits (R&D System, Minneapolis, MN, USA, and Thermo Fisher Scientific, Massachusetts, USA), according to the manufacturer instructions. BM serum levels of IL-17 and IL-23 were evaluated by multiplex cytokine assay kit (Bio-Plex^®^ Multiplex System, Biorad, California, USA). Soluble CX3CL1 levels were tested in BM plasma by ELISA (R&D System, Minneapolis, MN, USA).

Data from ELISA quantification were then correlated with flow-cytometry data and clinical parameters at diagnosis, International staging system (ISS), cytogenetic features and bone disease.

### Statistical Analysis

Comparison among the four groups was performed with the Kruskal-Wallis test and pairwise comparisons with the Mann-Whitney test or with parametric unpaired t-test. The Spearman test was used for correlations. A p-value <0.05 was considered significant. GraphPad Prism 8™ (GraphPad Software Inc., La Jolla, CA, USA) was used for all the statistical analysis.

## Results

### PD-L1 Expression on Both Plasma Cell and Monocyte Compartments Does Not Differ Between Patients With SMM and Active MM

**Figure 1:**
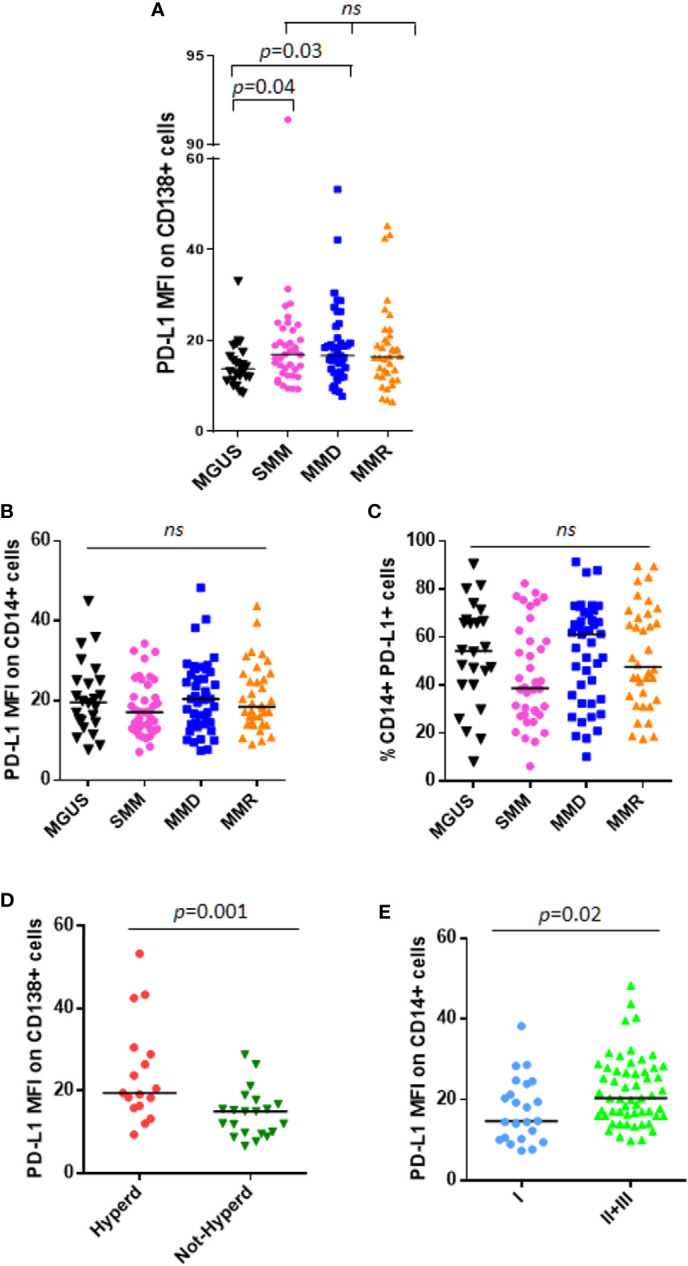
PD-L1 expression on PC and monocytes in patients with MGUS, SMM and active MM. Dot plots represent the median fluorescence intensity (MFI) of PD-L1 on CD138^+^ **(A)** and CD14^+^ **(B)** cells from BM aspirates of patients with MGUS (n = 24), SMM (n = 38), MMD (n = 43), MMR (n = 36) (*p* value calculated by Kruskal-Wallis test). **(C)** Dot plots represent the median BM %CD14^+^PD-L1^+^ cells of patients with MGUS, SMM and active MM. (*p* value calculated by Kruskal-Wallis test). Patients with MMD or MMR were classified based on ISS. **(D)** Hyperdiploidy was evaluated in 38 patients out of 79; dot plot represents the median PD-L1 MFI on CD138^+^ cells which is increased in patients with (Hyperd) as compared with patients without hyperdiploidy (Not-Hyperd) (*p* value calculated by Mann-Whitney test). Patients with MMD or MMR were classified based on ISS. **(E)** Dot plots represent the median PD-L1 expression on monocytes, which is increased in patients with advanced ISS (*p* value calculated by Mann-Whitney test). ns, not significant.

We analyzed PD-L1 expression on CD138^+^ cells and CD14^+^ cells from 24 patients with MGUS, 38 patients with SMM and 79 with active MM, including both MMD and MMR. In line with other studies (13), we considered the unimodal distribution of PD-L1 on PCs. We showed that PD-L1 expression on PC from MGUS patients is lower as compared with SMM and MMD patients (MGUS vs. SMM, median: 13.78 vs. 16.93, *p* = 0.04; MGUS vs. MMD, median: 13.78 vs. 16.70, *p* = 0.03, Mann-Whitney test) (**Figure 1A**). Interestingly, no differences were found in terms of PD-L1 expression intensity (median fluorescence intensity, MFI) on both PCs and monocytes between SMM and MM patients (**Figures 1A–C**).

Moreover, we did not find any correlation between PD-L1 expression and tumor burden (% of PC evaluated in the BM biopsy) (**Supplementary Figures 2A, B**).

SMM patients were also stratified according to both Mayo-Clinic and PETHEMA scores and PD-L1 distribution was analyzed in relation with this classification. However, we did not find any significant differences in terms of PD-L1 expression on both PCs and monocytes, among high, intermediate, and standard-risk patients (data not shown).

Afterwards, we performed analysis focusing on patients with active MM. Since PD-L1 gene is encoded on chromosome 9, we investigated a possible correlation between PD-L1 expression and the cytogenetic features, especially hyperdiploidy (evaluated in 38 patients out of 79), and we found that CD138^+^ cells from hyperdiploid patients express higher levels of PD-L1 as compared with not-hyperdiploid ones (19.46 vs. 14.99, Mann-Whitney test, *p* = 0.001) (**Figure 1D**). Classifying MM patients according to the ISS, we described a higher PD-L1 expression on CD14^+^ cells in patients with ISS = II and III as compared with ISS = I patients (ISS II + III vs. I, median MFI 20.35 vs. 14.72, Mann-Whitney test, p = 0.02) (**Figure 1E**), with no differences on PC compartment (data not shown).

Focusing on MMR subset, we evaluated the possible impact of the lines of treatments and the refractoriness on PD-L1 expression on both PCs and monocytes. However, both the number of therapeutic lines and the refractoriness to both IMiDs and proteasome inhibitors (PIs) did not show any effect on PD-L1 expression (data not shown).

### CD14^+^CD16^+^ Cells Express PD-L1 at Higher Intensity as Compared With Classical Monocytes CD14^+^CD16^−^

**Figure 2:**
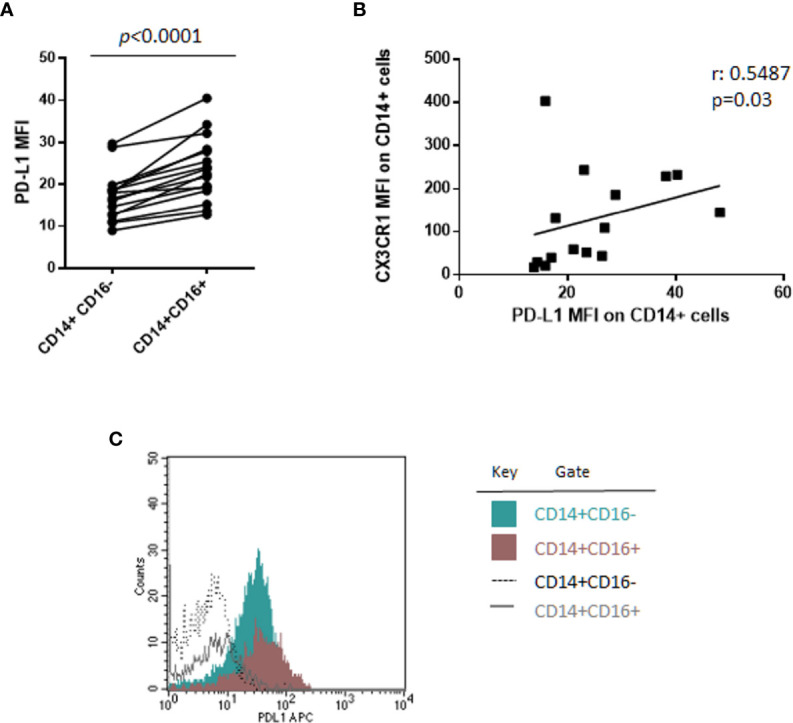
CD14^+^CD16^+^ cells express PD-L1 at higher intensity as compared with classical monocytes CD14^+^CD16^−^. **(A)** PD-L1 expression intensity on classical (CD14^+^CD16^−^) and non-classical (CD14^+^CD16^+^) monocytes in 16 patients with monoclonal gammopathies (SMM = 6, MMD = 4, MMR = 6). Paired samples are linked by a line. p-values between the indicated groups were calculated using the Wilcoxon signed-rank test. **(B)** Spearman’s correlation test between PD-L1 and CX3CR1 expression (MFI) on CD14^+^ monocytes. **(C)** Histogram shows flow-cytometry data of PD-L1 expression on classical CD14^+^CD16^−^ and non-classical CD14^+^CD16^+^ cells from a representative patient.

Of note, a sub-analysis of monocyte subsets on 16 patients, including 6 SMM and 10 MM patients, revealed, for the first time, that CD14^+^CD16^+^ cells express PD-L1 at higher intensity compared with classical monocytes CD14^+^CD16^−^ (median PD-L1 MFI, 23.09 vs. 17.41, Wilcoxon test, *p* < 0.0001) (**Figure 2A**), independently from the stage of disease. In addition, we previously demonstrated a higher expression of CX3CR1 in the non-classical monocyte subset in patients with MM (18); excitingly, in this study we found a positive correlation between CX3CR1 and PD-L1 expression on total monocytes (Spearman r: 0.5487, *p* = 0.03) (**Figure 2B**), thus suggesting a pro-inflammatory and immune suppressive profile of these cells in our cohort of patients. Flow-cytometry data from a representative patient are showed in **Figure 2C**.

### PD-1 Distribution and Expression on T Cell Compartments Are Similar Between Patients With Asymptomatic SMM and Active MM Disease

**Figure 3:**
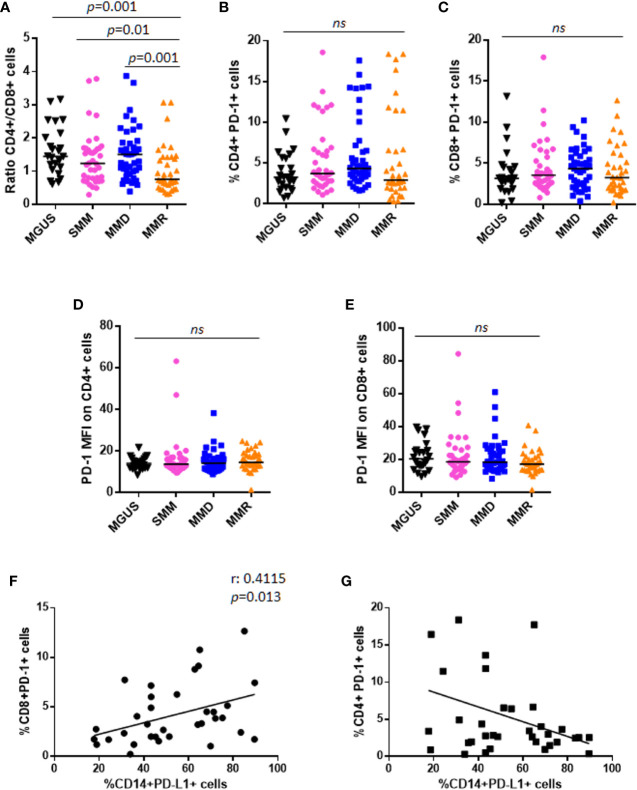
PD-1 expression and distribution on T lymphocytes in patients with MGUS, SMM and active MM. **(A)** Dot plots represent the CD4^+^/CD8^+^ ratio within BM niche of patients with MGUS (n = 24), SMM (n = 38), MMD (n = 43), MMR (n = 36) (*p* value calculated by Mann-Whitney test). Graphs represent individual values of %CD4^+^PD-1^+^ cells **(B)**, %CD8^+^PD-1^+^ cells **(C)**, evaluated on CD138^−^ fraction; and PD-1 expression intensity on both CD4^+^ **(D)** and CD8^+^ cells **(E)**, in patients with MGUS, SMM, MMD, and MMR. (ns, not significant). Analysis on relapsed MM patients revealed a positive correlation between CD14^+^PD-L1^+^% and CD8^+^PD-1^+^%, as indicated in the graph **(F)**. No significant correlation was found between CD14^+^PD-L1^+^% and CD4^+^PD-1^+^% **(G)**.

Both CD4^+^ and CD8^+^ T cells were analyzed by flow-cytometry in our cohort of patients with MGUS, SMM, and active MM. A significant decrease of CD4^+^/CD8^+^ ratio was observed in patients with MMR as compared with MGUS (0.75 vs. 1.45, Mann-Whitney test, *p* = 0.001), SMM (0.75 vs. 1.23, Mann-Whitney test, *p* = 0.01), and MMD (0.75 vs. 1.5, Mann-Whitney test, *p* = 0.001) (**Figure 3A**). Focusing on MMR subset, we evaluated the possible impact of the lines of treatments and the refractoriness on CD4^+^/CD8^+^ ratio. Our results indicated that patients who received ≥3 lines of treatments displayed a decreased CD4^+^/CD8^+^ ratio as compared with patients treated with 1 or 2 therapeutic lines (data not shown). However, no differences in CD4^+^/CD8^+^ ratio emerged among patients treated with different therapeutic agents (IMiDs, PIs) (data not shown).

Moreover, no differences were reported in terms of CD4^+^PD-1^+^%, CD8^+^PD-1^+^%, and their respective PD-1 expression intensity, among patients with MGUS, SMM, and active MM (Kruskall-Wallis test) (**Figures 3B–E**). Similar to PD-L1 expression, we did not find any correlation between PD-1 expression on T cells and PC% in the BM biopsy (**Supplementary Figures 2C–F**).

PD-1 distribution was also analyzed in relation with Mayo-Clinic and PETHEMA scores systems in SMM patients. However, we did not find any significant differences in terms of PD-1 expression on both CD4^+^ and CD8^+^ cells, among high, intermediate, and standard-risk patients (data not shown).

Interestingly, analysis on MMR patients revealed a positive correlation between CD14^+^PD-L1^+^% and CD8^+^PD-1^+^% (Spearman r: 0.4115, *p* = 0.013), but not CD4^+^PD-1^+^% (**Figures 3F, G**).

As for PD-L1, we performed analysis focusing on patients with active MM and we showed that patients with ISS = II and III have higher CD8^+^PD-1^+^% (II + III vs. I, 4.42 vs. 2.48, Mann-Whitney test, *p* = 0.007), but not CD4^+^PD-1^+^% (II + III vs. I, 3.91 vs. 3.79, Mann-Whitney test, *p* = 0.462), as compared with ISS = I patients (**Supplementary Figures 3A, B**).

Stratifying MM patients according to the cytogenetic risk (17), we observed an increased PD-1 expression on CD4^+^ cells (median MFI 15.54 vs. 13.7, Mann-Whitney test, p = 0.02) of patients with SR as compared with HR ones (**Supplementary Figures 3C, D**).

Since it has been reported by An et al. (19) that PD-L1 expression increases during osteoclastogenesis and mature osteoclasts induce PD-L1 expression on MM cell lines, we performed the analysis of PD-L1/PD-1 distribution by splitting MM patients into osteolytic and not osteolytic, according to the presence or absence of bone disease. However, we found no differences (**Supplementary Figures 4A–E**) except for CD8^+^PD-1^+^%, which was higher in not-osteolytic patients compared with osteolytic ones, even without reaching a statistical significance (5.73 vs. 3.87, Mann-Whitney test, *p* = 0.071) (**Supplementary Figure 4F**).

### PD-L1 Expression on Monocytes and CD8+PD-1+% Are Inversely Correlated With BM IL-27 Serum Levels

Results from flow-cytometry analysis were then correlated with measurements of BM serum levels of several cytokines known to regulate PD-L1 expression or to exert pro/anti-tumor activity in MM (20). Specifically, we tested INF-γ levels, known to *in vitro* upregulate PD-L1 expression on both PC and monocytes (11, 21). However, we found very low IFN-γ levels in sera from patients with SMM or MM, with no differences based on diagnosis (data not shown). In addition, we did not report any correlation between PD-L1 expression on either CD138^+^ cells or CD14^+^ cells and IFN-γ levels *in vivo* (**Supplementary Figures 5A, B**). On the other hand, we confirmed *in vitro* up-regulation of PD-L1 on both PCs and monocytes after INF-γ (100 UI/ml) treatment of total BM MNCs from 3 MM patients (**Supplementary Figures 5C, D**).

**Figure 4:**
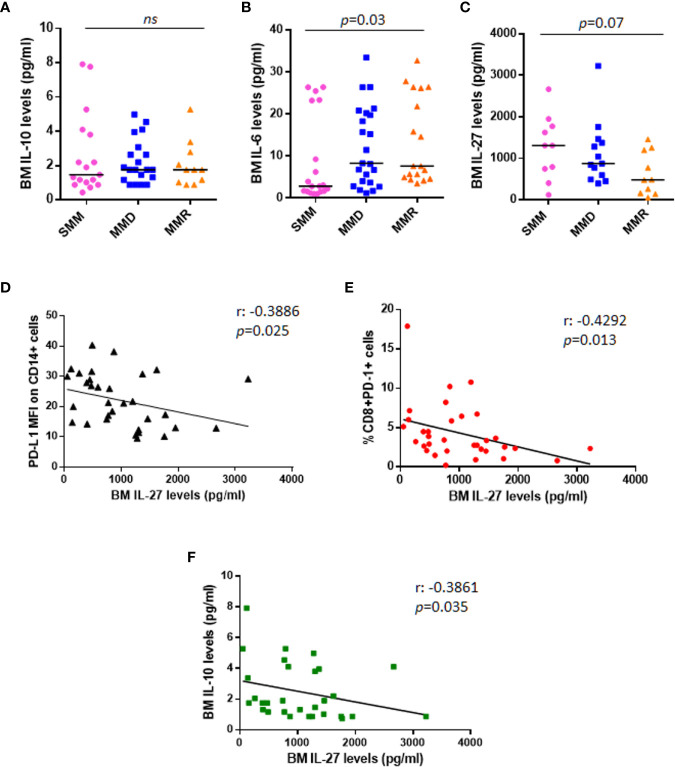
Anti-tumoral IL-27 BM serum levels are inversely correlated with PD-L1 expression on monocytes and %CD8^+^PD-1^+^ cells. Dot plots represent median BM serum levels of IL-10 **(A)**, IL-6 **(B)**, and IL-27 **(C)** measured by ELISA in a subgroup of patients at different stages of disease (*p* values calculated by Mann-Whitney test). **(D)** The graph shows a significant inverse correlation (r: −0.3886) between PD-L1 MFI on CD14+ cells and IL-27 BM serum levels in a total cohort of 33 patients with SMM and active MM. **(E)** IL-27 BM serum levels are inversely correlated with %CD8^+^PD-1^+^ cells (r: −0.4292). **(F)** Inverse correlation between anti-tumoral IL-27 levels and immune-suppressive IL-10 is shown in the graph (r: −0.4292). (*p* values calculated by Spearman’s correlation). ns, not significant.

Based on literature data on IL-10 effect on PD-L1 expression in monocytes (21, 22), we measured BM sera levels of IL-10 in our cohort of patients. Our data show no differences among patients at different stages of disease (**Figure 4A**); in addition, IL-10 BM levels did not correlate with PD-L1 expression on both PCs (Spearman r: 0.102, *p* = 0.49) and monocytes (Spearman r: 0.181, *p* = 0.22) (data not shown).

We also checked BM levels of the main MM pro-survival cytokine, IL-6, which is known to be higher in MM patients as compared with HDs (23); our data confirmed the increased BM IL-6 levels in patients with advanced stage of disease (SMM vs. MMD vs. MMR pg/ml: 2.830 vs. 8.270 vs. 7.625, Kruskal-Wallis test, *p* = 0.03) (**Figure 4B**); however, we did not find any correlation with PD-L1/PD-1 expression on PCs (Spearman r: 0.042, *p* = 0.77) and BM microenvironment cells (data not shown). We then measured BM levels of IL-27, with demonstrated anti-tumor activity in MM (24), in 33 patients, including 10 SMM, 13 MMD, and 10 MMR. In line with its role in MM, we found that IL-27 levels progressively decrease in the advanced stages of disease, even without reaching a statistical significance (SMM vs. MMD vs. MMR pg/ml: 1303 vs. 870.1 vs. 478.7, Kruskal-Wallis test, *p* = 0.07) (**Figure 4C**).

Interestingly, the anti-tumoral IL-27 BM serum levels inversely correlated with PD-L1 MFI on CD14^+^ cells (Spearman r: −0.3886, *p* = 0.025) (**Figure 4D**), with CD8^+^PD-1^+^% (Spearman r: −0.4292 *p* = 0.013) (**Figure 4E**) and with the immunesuppressive cytokine IL-10 serum levels (Spearman r: −0.3861, *p* = 0.035) (**Figure 4F**), independently from the stage of disease.

Lastly, we measured IL-17 and IL-23 BM levels by multiplex cytokine assay kit; however, data were undetectable for the majority of the analyzed samples.

## Discussion

In the last decade, immunotherapy, including PD-L1/PD-1 immunecheckpoint blockade, has emerged as a promising therapeutic strategy in the treatment of different solid tumors and some hematological malignancies as Hodgkin lymphoma (4). However, the role of mAbs against PD-L1/PD-1 immunecheckpoint is still debated in the treatment of MM patients. Several *in vitro* studies demonstrated the ability of anti–PD-1 mAbs to restore T cells and NK cells functions inhibited by the presence of MM cells (5, 11, 25–27). In addition, Dhodapkar et al. revealed that PD-L1 expression on tumor and infiltrating immune cells correlates with the risk of malignant transformation into clinical MM (28); accordingly, Tamura et al. observed clinical progression in patients with higher PD-L1 expression on PCs (12), thus suggesting a role of PD-L1/PD-1 axis in MM immune escape.

However, no clinically meaningful results emerged from early clinical studies on PD-1/PD-L1 inhibitors as single agents in relapsed/refractory MM (RRMM) patients (9, 10); furthermore, FDA put on hold all the clinical trials using anti–PD-1/PD-L1 antibodies in combination with other therapeutic agents, as the IMiDs, in MM because of severe adverse events (https://www.fda.gov/Drugs/DrugSafety/ucm574305.htm).

It thus remains a major challenge for this therapeutic strategy to identify the most susceptible patient subset and avoid serious toxicities and unnecessary costs for non-responders.

It also lacks a scientific *consensus* on PD-1/PD-L1 expression in MM cells and BM niche cells (7, 8, 11–14). Of note, the majority of the studies compared MM patients with MGUS or HDs, while few data have been reported on asymptomatic patients with SMM (15). In our study, in line with Paiva et al. (13), we considered the unimodal distribution of PD-L1 on PCs rather than focusing on the percentage of PD-L1^+^ PCs as the majority of the studies (7, 8). Moreover, results on PD-L1/PD-1 expression on monocytes and T cells were normalized for PCs infiltration and thus evaluated as % on BM CD138^−^ fraction. We observed a high heterogeneity among samples belonging to the same disease category; however, our data interestingly showed that PD-L1 expression is lower in the first asymptomatic stage, MGUS, as compared with the advanced stages of SMM and active myeloma. More excitingly, neither PD-L1 expression on CD138^+^ cells and monocytes nor PD-1 on CD4^+^ or CD8^+^ cells significantly differ between SMM and MM patients. All these data plead for the use of PD-L1/PD-1 blockade in the asymptomatic SMM patients, but not in the very early stage MGUS, as compared with relapsed myeloma patients.

Our analysis on monocytes also revealed, for the first time, that non-classical CD14^+^CD16^+^ cells express PD-L1 at higher intensity compared with classical monocytes CD14^+^CD16^−^, independently from the stage of disease. These results are in line with recent findings from Bianchini et al. (29) who described PD-L1 as a selective marker for non-classical monocytes under inflammatory conditions. In addition, we found a positive correlation between CX3CR1, known to be higher in the non-classical monocyte subset in patients with MM (18), and PD-L1 expression on total monocytes, thus suggesting a pro-inflammatory and immune suppressive profile of these cells in our cohort of patients. However, further studies are needed to better define the relationship between these two molecules in patients with monoclonal gammopathies.

Focusing on patients with active myeloma, we analyzed flow-cytometry data in relation with ISS and cytogenetic features of our cohort of patients. We observed that patients with ISS = II and III show higher PD-L1 expression on CD14^+^ cells and higher CD8^+^PD-1^+^% as compared with ISS = I patients, in line with results from Chang et al. (30). Patients were then stratified according to Sonneveld criteria (17), adopted in the majority of the recent studies where GEP analysis were not available to use the updated Mayo Clinic stratification method. In agreement with data from Gorgün et al. (7), we found that PD-L1 is expressed at higher levels in hyperdiploid patients. However, PD-L1 expression is similar in patients with high and standard cytogenetic risk (data not shown). Overall these data suggest that the expression of this immune checkpoint does not contribute as a HR factor in active myeloma disease. Since bone disease is the hallmark of MM and An et al. (19) reported that PD-L1 expression increases during osteoclast formation which in turn induces PD-L1 expression on MM cell lines, we correlated flow-cytometry data with the presence or absence of bone disease in our cohort of patients. However, our results do not indicate a correlation between PD-L1/PD-1 distribution and the presence of osteolysis, except for CD8^+^PD-1^+^%, which was higher in not-osteolytic patients compared with osteolytic ones, even without reaching a statistical significance. This result will be further investigated in a larger cohort of patients in future studies.

Focusing back to PD-L1/PD-1 distribution among patients at different stages of disease, the similarities between patients with asymptomatic and active myeloma, together with the highly compromised BM microenvironment of relapsed MM patients, potentially suggest that SMM patients could benefit from PD-L1/PD-1 blockade, more than MMR which are currently the main candidates for anti-PD-L1/PD-1 treatment. Indeed, it should be considered that MMR patients display a higher tumor burden and are heavily pre-treated. In addition, based on our results, they show an inverted CD4^+^/CD8^+^ ratio along with high levels of pro-tumoral IL-6 and a positive correlation between CD14^+^PD-L1^+^% and CD8^+^PD-1^+^% cells, thus indicating a highly compromised immune compartment with a low amount of effector CD4^+^ cells, which could explain the lack of efficacy of PD-L1/PD-1 blocking antibodies. In addition, it is known that CD8^+^ T cells from myeloma patients co-express other immune checkpoints, such as LAG-3, TIM-3, CTLA4, and TIGIT (31–33) suggesting multiple pathways limiting T-cell responses at this stage of disease and thus explaining the lack of effect from PD-L1/PD-1 blockade as monotherapy. Remarkably, a defective immunosurveillance allows for the persistence and proliferation of MM cells, and the intimate interaction among MM microenvironment gatekeepers, as endothelial cells, MM cells and immune cells creates a permissive niche that allows undisturbed cancer proliferation. The niche and the gatekeepers thus represent an additional immune regulatory mechanism that inhibits the development of antitumor immunity and may impair the success of cancer immunotherapy in these patients.

On the other hand, SMM patients could be the best subset of patients for the use of immunecheckpoint inhibitors as further supported by the results from Dhodapkar et al., on the correlation between PD-L1 expression and the risk of disease progression which suggest that PD-L1 blockade in early stages of disease could prevent MM progression (28). In addition, recent data from Bar et al. (34) on a pilot trial of single agent anti-PD-L1, atezolizumab, in asymptomatic MM patients, revealed a specific *in vivo* rapid expansion and activation of myeloid cells, and T cell reinvigoration after treatment. Moreover, *in vitro* experiments from the same study showed that PD-L1 blockade enhances DC functions (34). Interesting results, including a complete response, have been also reported from a pilot study on a SMM patient treated with anti–PD-1 mAb, pembrolizumab (35). All over these data thus support the potential clinical activity of PD-L1/PD-1 blockade as single agents in patients with early stage of myeloma.

In order to find other markers, which could allow to predict the clinical responsiveness to PD-L1/PD-1 checkpoint blockade, we also measured BM serum levels of different soluble factors known to regulate PD-L1 expression or to exert pro/anti-tumor activity in MM; we then analyzed the results in relation with PD-L1/PD-1 expression on PC and BM niche cells.

Despite literature data describe IFN-γ as the main factor inducing PD-L1 *in vitro* up-regulation on both PCs and monocytes (11, 21), we did not find any significant correlation with PD-L1 distribution in our cohort of patients. Conversely, PD-L1 up-regulation on PCs and monocytes was detected after *in vitro* treatment of BM MNCs with IFN-γ; these data thus suggest that *in vitro* data do not reflect *in vivo* effects of this cytokine in BM niche, possibly due to a dilution of IFN-γ in the sera and suggesting the use of alternative methods to quantify this cytokine in the focal lesion of MM patients.

We then analyzed BM levels of IL-6, the main MM pro-survival factor (36), which is also involved in PD-L1 up-regulation on monocytes (37) and recently described as predictor of response to PD-L1/PD-1 inhibitors in NSCLC patients (38). Our data confirmed higher IL-6 BM levels in patients with active myeloma compared with SMM, however no correlation emerged with PD-L1/PD-1 expression. On the other hand, we found an inverse correlation between BM levels of IL-27 and PD-L1 expression on monocytes and %CD8^+^PD-1^+^ cells, independently from the stage of disease. These results are in line with IL-27 anti-tumoral activity in MM (24); despite the recent findings on its role in PD-L1 *in vitro* up-regulation in different solid cancer cells (39).

## Conclusions

To conclude, our data indicate that there are no differences between the PD-L1/PD-1 immune profile of patients with asymptomatic SMM as compared with active MM. This suggests that SMM patients could be an interesting target for PD-L1/PD-1 inhibition therapy, in light of their less compromised and more responsive immune compartment, as demonstrated by their cytokine profile with low levels of IL-6 and high amount of IL-27 and the normal CD4^+^/CD8^+^ ratio.

It is thus encouraging to design clinical trials on PD-L1/PD-1 inhibitors as single agents in patients with asymptomatic myeloma.

## Data Availability Statement

The original contributions presented in the study are included in the article/**Supplementary Material**. Further inquiries can be directed to the corresponding authors.

## Ethics Statement

The studies involving human participants were reviewed and approved by Comitato Etico per Parma, Via Gramsci 14-43126 Parma. The patients/participants provided their written informed consent to participate in this study.

## Author Contributions

FC collected and processed the samples, supported by PS, VM, and DT. RV and FC performed the flow cytometry analysis. LN and BDP provided clinical data and enrolled patients. FC and VM performed ELISA assay. GS performed cytogenetic analysis. FC and NG analyzed data and wrote the manuscript. NG and JB-G read and provided comments, and NG approved the final version of the manuscript. All authors contributed to the article and approved the submitted version.

## Funding

This work was supported in part by a grant from the Associazione Italiana per la Ricerca sul Cancro IG2017 n. 20299, the International Myeloma Foundation under 2018 Brian D. Novis Senior Research Grant and a grant from the Ministero della Salute Italiana PE-2016-02361261.

## Conflict of Interest

NG received research funding and honoraria from Amgen, Bristol Mayers Squibb, Celgene, Millenium Pharmaceutical, and Janssen Pharmaceutical.

The remaining authors declare that the research was conducted in the absence of any commercial or financial relationships that could be construed as a potential conflict of interest.

## Abbreviations

BM, Bone marrow; DC, Dendritic cell; F, female; h, Hours; HDs, healthy donors; HR, high risk; IMiDs, Immunomodulatory drugs; ISS, International stage system; M, male; MFI, Median fluorescence intensity; MGUS, Monoclonal gammopathies of undetermined significance; MNCs, Mononuclear cells; MM, Multiple myeloma; MDSCs, Myeloid derived suppressor cells; NK, Natural killer; MMD, Newly diagnosed MM; NSCLC, Non-small-cell lung cancer; PIs, Proteasome inhibitors; PC, Plasma cells; PD, Programmed cell death protein; MMR, Relapsed MM; SMM, Smoldering MM; Th, T helper.
