# Supplementary material for: PD-L1/PD-1 Pattern of Expression Within the Bone Marrow Immune Microenvironment in Smoldering Myeloma and Active Multiple Myeloma Patients
Source: Front Immunol. 2021 Jan 8;11:613007. doi: 10.3389/fimmu.2020.613007 (PMC7820813; doi:10.3389/fimmu.2020.613007)
Supplement: Supplementary file 1 [file DataSheet_1.docx]

**SUPPLEMENTARY DATA**

**
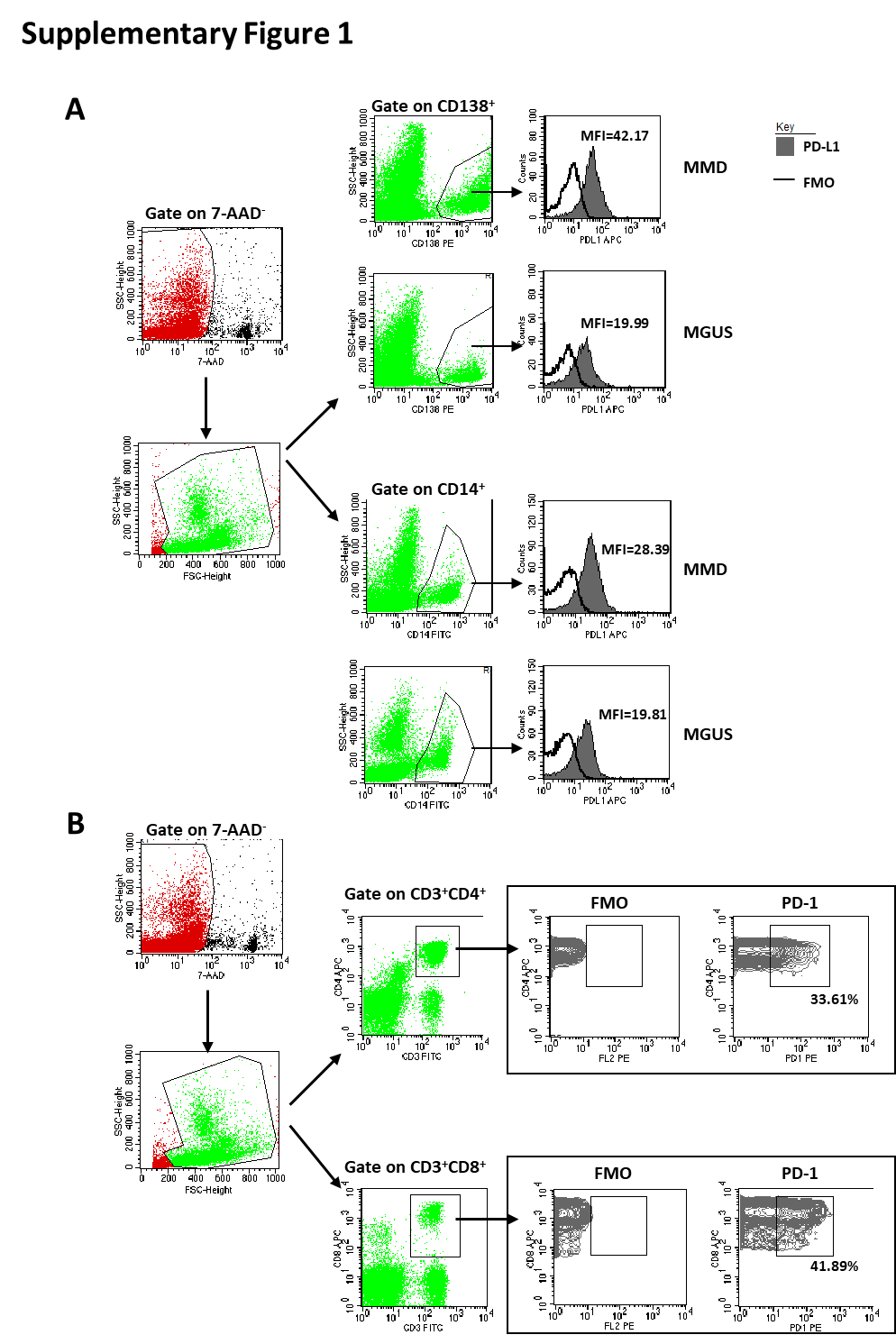
Supplementary Figure 1**

**Supplementary Figure 2**

**
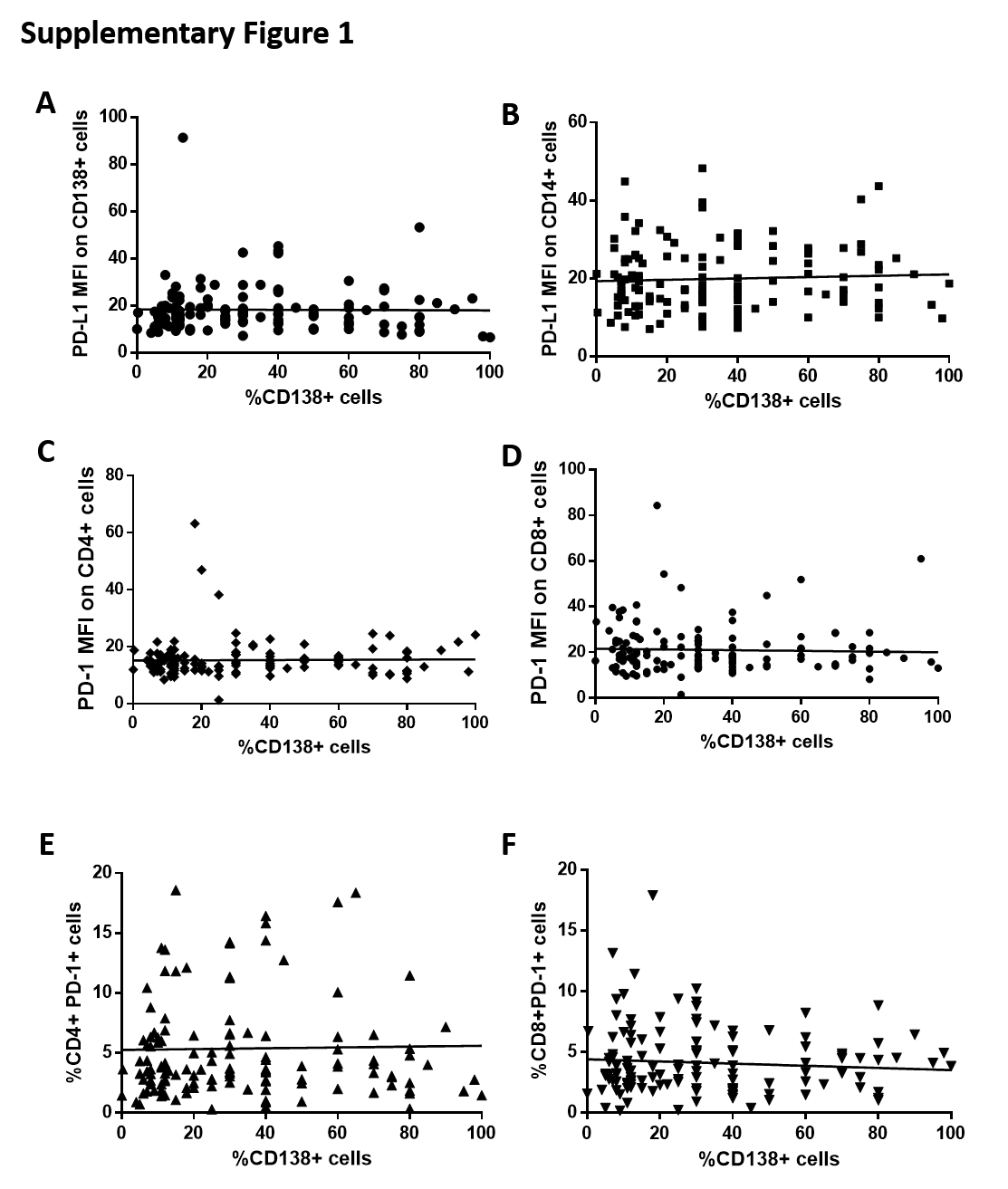
**

**Supplementary Figure 3**

**
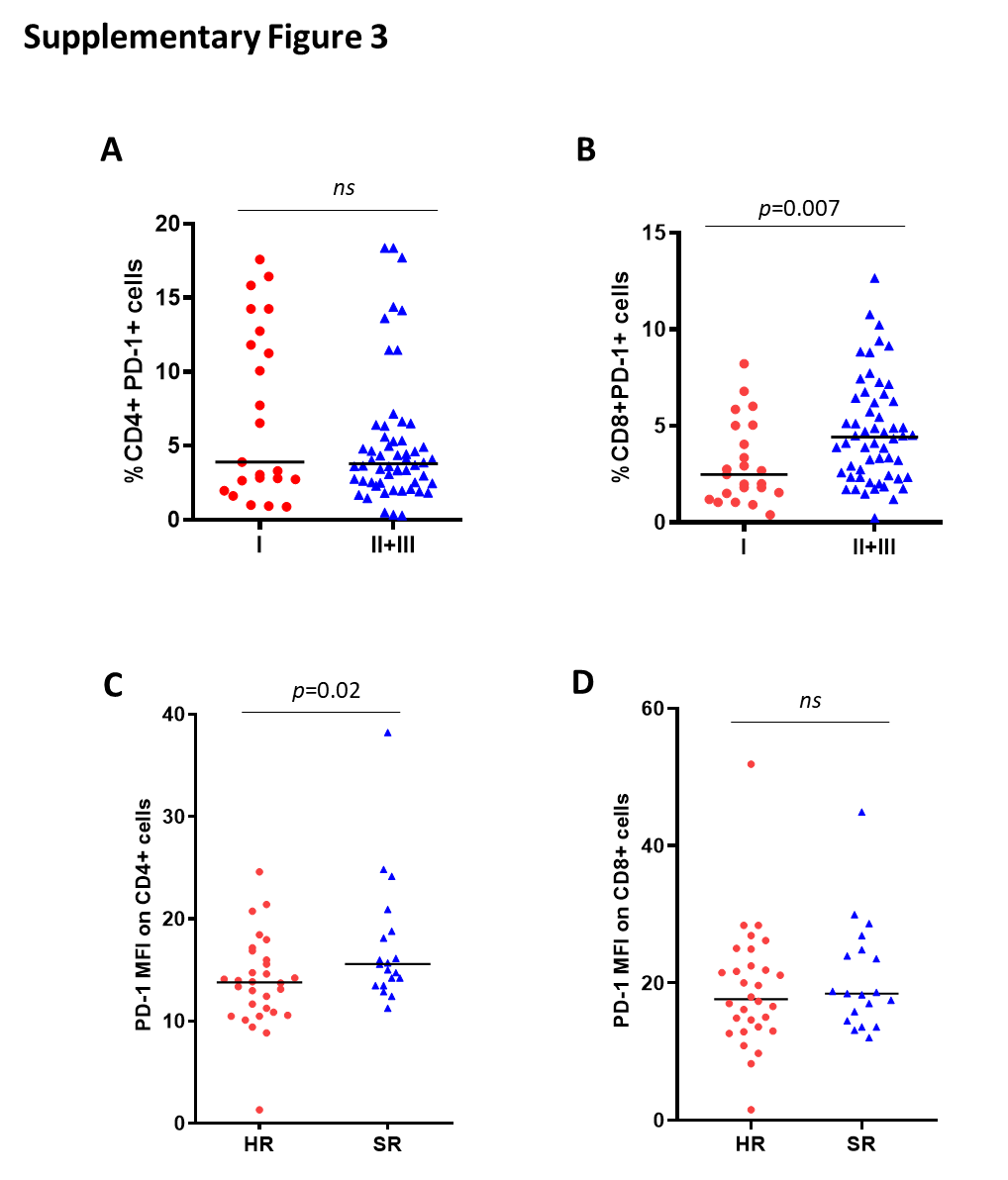
**

**
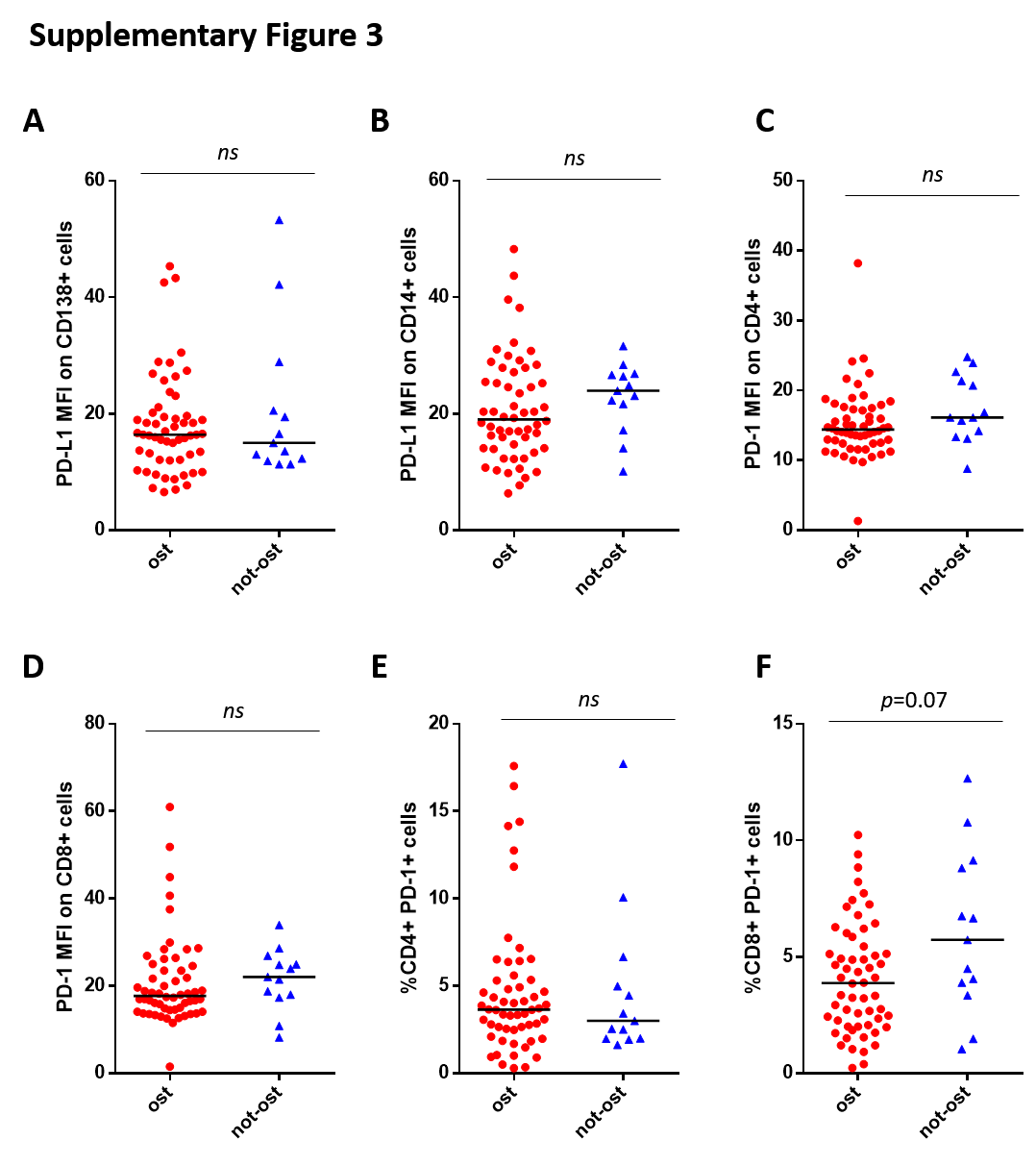
Supplementary Figure 4**

**
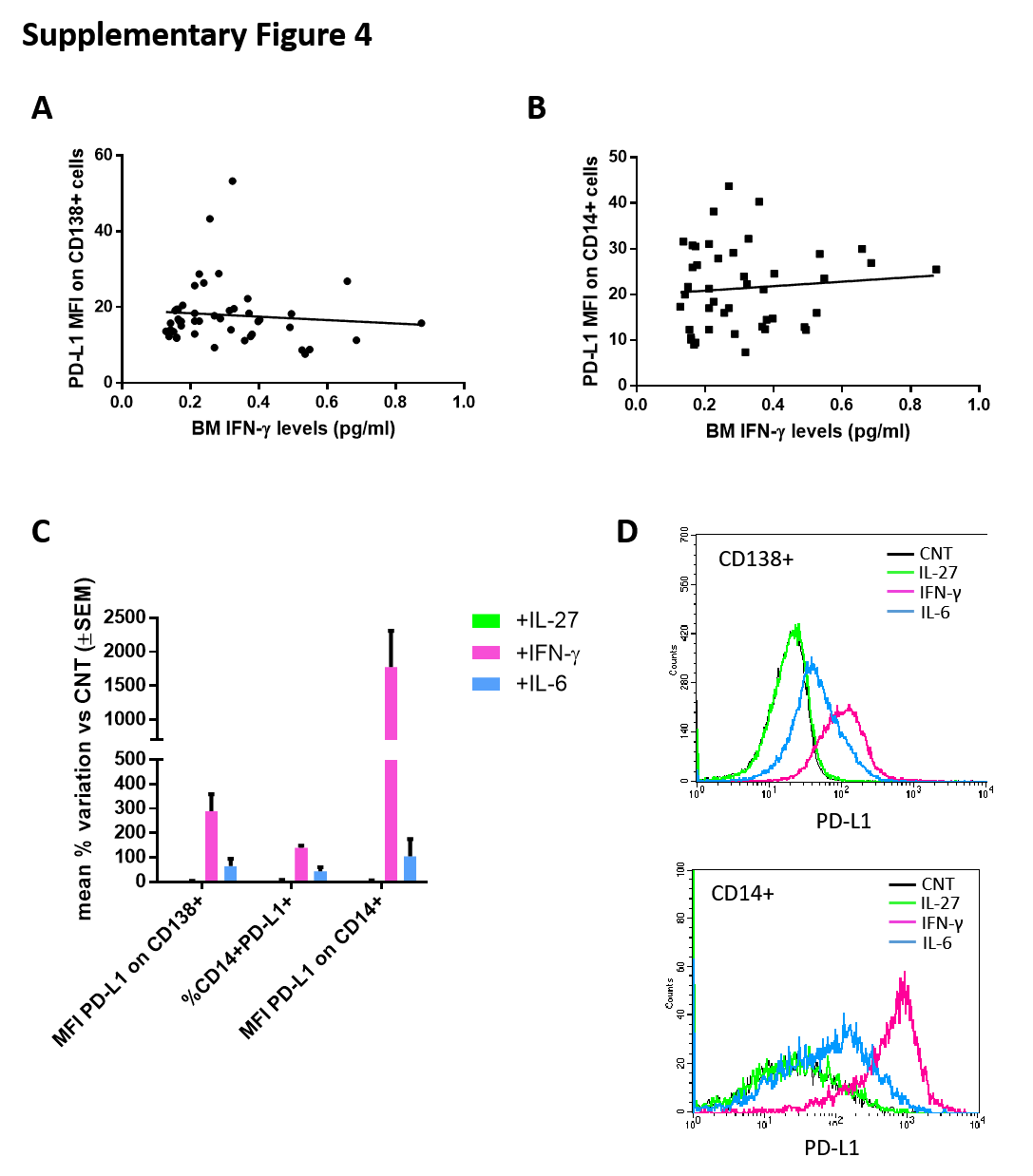
Supplementary Figure 5**

**LEGENDS OF SUPPLEMENTARY FIGURES**

**Supplementary Figure 1: Representative gating strategy for defining PD-L1^+^ and PD-1^+^ cell subsets.**

**(A)** The analysis includes a first live gating (7-AAD- events, R1) and a subsetting forward and side scatter gating (R2) to identify cells, while removing debris and cell fragments. MM cells are identified as CD138+ (top); total monocytes are defined as CD14+ (bottom). The histogram plots on the right show the PD-L1 MFI evaluated on the corresponding gated cell populations, from 1 MMD and 1 MGUS patient.

**(B)** The analysis includes a first live gating (7-AAD- events, R1) and a subsetting forward and side scatter gating (R2) to identify cells, while removing debris and cell fragments. T cell subsets are identified as CD3^+^CD4^+^ (top) and CD3^+^CD8^+^ (bottom) cells. The contour plots on the right show the % of PD-1+ cells among CD3^+^CD4^+^ and CD3^+^CD8^+^ cells. Fluorescence minus one (FMO) controls were used to set PD-1 gates.

**Supplementary Figure 2: PD-L1/PD-1 pattern of distribution is not correlated with tumor burden in patients with SMM and active MM.**

Graphs show the absence of correlation between tumor burden (expressed as %CD138+ cells in bone biopsies) and PD-L1 expression on PC **(A)** and monocytes **(B)**, PD-1 expression on CD4+ cells **(C)** and CD8+ cells **(D)**, and their respective percentages **(E, F)** in patients with MGUS, SMM and active MM.

**Supplementary Figure 3: PD-1 expression is increased in patients with advanced ISS and low cytogenetic risk.**

Patients with MMD or MMR were classified based on ISS. Dot plots represent the median %CD4^+^PD-1^+^ **(A)** and %CD8^+^PD-1^+^ **(B)** in patients with ISS=I compared with advanced ISS=II and III patients. Patients were also classified based on cytogenetic risk (presence/absence of del(17p) and/or IGH translocations and/or gain(1q) and/or del(1p): high risk, HR/standard risk, SR). Patients with SR show increased PD-1 expression on CD4^+^ cells **(C).** PD-1 expression on CD8^+^ cells did not change between the two groups **(D).** (*p* value calculated by Mann-Whitney test).

**Supplementary Figure 4: PD-L1/PD-1 profile in relation with bone disease.**

MM patients were classified into osteolytic (ost) and not osteolytic (not ost), according to the presence or absence of bone disease. Dot plots show individual data for PD-L1 expression on PC **(A)** and monocytes **(B),** PD-1 MFI on CD4^+^ **(C)** and CD8^+^ **(D)** cells, and the respective % of PD-1^+^ cells **(E, F).** (*p* value calculated by Mann-Whitney test). (ns: not significant)

**Supplementary Figure 5: IFN-γ BM levels are not correlated in-vivo with PD-L1 expression on PC and monocytes in BM niche of patients with SMM and active MM.**

IFN-γ levels were measured in BM serum of 42 patients, including 10 SMM, 21 MMD and 11 MMR, by ELISA. The lack of correlation between IFN-γ levels and PD-L1 MFI on both PC **(A)** and monocytes **(B)** *in vivo*, is displayed in the graphs. BM-MNCs from 3 MM patients were treated *in vitro* with IFN- γ (100 UI/ml), IL-6 (20 ng/ml) and IL-27 (50ng/ml) for 96 h. PD-L1 expression was then checked by flow-cytometry. **(C)** Bar-graph shows the mean % variation ± SEM of PD-L1 expression (MFI) on both PC and monocytes, in the presence of IFN-γ, IL-6 and IL-27 as compared with untreated cells (CNT), from 3 independent experiments. **(D)** Flow-cytometry histograms from one representative experiment, showing the increase of PD-L1 expression on both PC (top) and monocytes (bottom), after IFN-γ *in vitro* treatment.
